# The Impact of Simulation-Based Training in Cardiovascular Medicine: A Systematic Review

**DOI:** 10.7759/cureus.50414

**Published:** 2023-12-12

**Authors:** Anthony G Kweki, Mohammad Sarwar Khan Tharin, Victor Baptista, Echofa Kenneth, Francis Rohin, Mark Scoote, Adam Q Howard

**Affiliations:** 1 Internal Medicine/Cardiology, Colchester Hospital, East Suffolk and North Essex NHS Foundation Trust (ESNEFT), Colchester, GBR; 2 Surgery, Colchester Hospital, East Suffolk and North Essex NHS Foundation Trust (ESNEFT), Colchester, GBR; 3 Anaesthesiology, Delta State University Teaching Hospital, Oghara, NGA; 4 Vascular Surgery, Colchester Hospital, East Suffolk and North Essex NHS Foundation Trust (ESNEFT), Colchester, GBR

**Keywords:** simulation-based teaching, apprentice, sytemic review, cardiovascular, medical education, simulation medicine

## Abstract

Cardiovascular medicine and practice in recent times have evolved as complex procedures are performed to manage difficult cases. The majority of these interventions are done percutaneously in order to minimize patient risk. Additionally, training specialist in handling these interventions require a lot of exposure to them; as such, patients are at higher risk of errors and complications from trainees before attaining expertise. In order to avoid these possible risks to patients and ensure their safety, using simulation commonly in cardiovascular specialist education is a possible trend in the future. This article aims to review randomized controlled trials that were performed in cardiology and vascular medicine regarding the use of simulating models to transfer skills to trainees. This study is a systematic review that includes publications dated from 2010 from any country and only in English. The search involved several combinations of search terms from medical subject headings (MeSH). Keywords in the title, abstract, and text for the population, intervention, control, and outcomes were first done in a pilot search to establish the sensitivity of the search strategy. Studies were searched in PubMed, Medline, Cochrane Library, Embase, CINAHL, and Hirani. Data were presented in the PRISMA flowchart and tabular form. A total of 389 studies were obtained from five databases using the search strategies. Eighty-nine studies were excluded for duplication. The total number of studies that did not meet the inclusion criteria was 269, and they were excluded based on abstract and title screening. Another 18 studies were excluded based on full-text screening. In this study, 13 articles were selected ranging from 2011 to 2022. The majority of the outcomes of the study demonstrated that simulation tutoring complements traditional methods of training. Countries of publication were the United States of America, Canada, Italy, Korea, California, Ireland, Germany, Belgium, Switzerland, United Kingdom, Netherlands, and France. Procedures simulated include coronary angiography, transseptal catheterization, cardiopulmonary resuscitation, ultrasound-guided radial artery cannulation, diagnostic angiograms, coiled carotid terminus aneurysms in the setting of subarachnoid hemorrhage, middle cerebral artery embolectomies, renal artery angioplasty/stenting, endovascular aneurysm repair, transvenous pacing wire, intra-aortic balloon pump, and pericardiocentesis. Despite the accredited drawback of availability and cost noted with simulation-based education, there is evidence that it offers many advantages compared to traditional teaching methods. From this study, simulation-based teaching has been shown to effectively transfer skills to trainees especially when used as an adjunct to the apprenticeship method. As a result, we recommend that virtual reality education should be integrated with real-life teaching in modern cardiovascular modules as this will help ensure early skill transfer while maintaining patient safety.

## Introduction and background

Simulation is a model of the dynamics of a real-world process or system. It offers trainees the opportunity to acquire practical knowledge and skills not just by studying theoretically but also by actively participating in hands-on activities, mimicking real-life situations [1]. In our contemporary healthcare system, prioritizing patient safety and mitigating medical errors are key areas of interest; as such, simulation-based training has emerged as a pivotal component of medical education [2]. This form of training affords high-quality instruction while ensuring a completely safe environment for patients [3]. Training in cardiovascular medicine seems well-suited for simulation-based practice; this attests to the fact that understanding and mastering techniques is highly required. Several very sophisticated procedures, such as coronary angioplasty with stenting, pacing, electrophysiological studies, transcatheter valve placement, and peripheral vascular interventions, have been the mainstay of teaching in cardiovascular medicine; howbeit, it is essential that these skills and techniques are taught and more cardiologists should be able to perform the procedures worldwide [4].

Simulation training has been employed in different specialties of medicine; the first recorded instance of incorporating simulation in healthcare took place in 1960 when a team of anaesthesiologists collaborated with a toy company to create a life-size simulator for a patient experiencing cardiac arrest [2]. Medical simulation has garnered a significant amount of attention as a result of technological advancements with a notable decrease in training duration and enhanced procedural intricacy [5]. Simulation training is considered a favorable approach toward offering education that effectively bridges theoretical knowledge and practical application [3].

Various complementary simulation methods have been applied in the field of cardiology [2]. Task trainer simulation models involve the creation of a simple, standardized teaching model designed to help learners practice specific skills, and the simulators represent a part of the body (e.g., thorax) for training in a particular skill [6]. Human patient simulators (HPSs) are an alternative and one of the most current instructing modalities in health education, and they involve the use of HPSs such as manikin designed to react physiologically as if the product were a real-life patient. The organs of these medical simulators, including their heart, blood vessels, lungs, and gastrointestinal tract, can also be made to respond to a variety of human interventions during clinical simulation engagements [7]. Scenarios with debriefing at a team level, standardizing patients, and virtual reality have also been reported in simulation studies [2,6].

Deuchler et al. [8] conducted a study on the clinical efficacy of simulated vitreoretinal surgery to prepare surgeons for the upcoming intervention in the operating room, and they aimed to evaluate the efficacy of the virtual reality training simulator, as well as prepare surgeons for performing pars plana vitrectomies, and its potential to predict the surgeons’ performance. Their outcome comparing each surgeon’s performance with and without warmup training showed a positive effect of warmup training on their competence in the operating room. Despite the large variation of conditions, the effect of warmup training on performance in the operating room was found to be statistically significant (p = 0.0302). Therefore, simulator training can serve as a warmup to increase the average performance.

In a bid to evaluate whether simulation-based training with a vessel phantom improves the basic skills of a novice required for ultrasound-guided radial artery cannulation in real patients, Oh et al. [9] conducted an assessor-blinded randomized controlled study where 21 anesthesiology residents were randomized into either a simulation group (n = 11) or control group (n = 10). Their findings reported that the first attempt success rate and dynamic needle-tip positioning ability using an ultrasound transducer were significantly higher in the simulation group than in the control group (81.8% vs. 50%, p = 0.002 and 68.2% vs. 7.5%, p < 0.001, respectively). Simulation-based training using a vessel phantom has effectively improved the first-attempt success rate for ultrasound-guided radial artery cannulation in real patients and the dynamic needle-tip positioning ability using ultrasound transducer.

Lee et al. [10] in a randomized controlled trial of simulation training in teaching coronary angiography, aimed to test whether structured simulation training, in addition to traditional methods, would improve coronary angiography image interpretation skills in a heterogeneous group of medical trainees. They recruited 105 subjects comprising of 20 medical students, 68 residents, and 17 fellows. They were randomized in a stratified fashion into a simulation training group that received simulation training, in addition to didactic teaching (n = 53), and a control training group that received didactic teaching alone (n = 52). Subjects in the simulation training arm had a higher delta score compared to the control (5.4 ± 4.2 versus 3.8 ± 3.7, p = 0.04). This trial demonstrated that simulation training complements traditional methods to improve coronary angiography interpretation skills.

Relevance of the study

Simulation-based training has been recognized as an adjunct to the apprenticeship paradigm of training in cardiovascular medicine, offering benefits such as metric-based assessments, and an opportunity to improve trainee-based outcomes [11]. Due to the possible risk to patient safety in the real-world experience procedure during training and trainers trying to meet up with targets, trainees tend to have limited procedure access. This is also true of complex cases that may be handled by the teachers in order to minimize patient risk. As a result, trainees are not adequately equipped for real-world practice with the relevant skills. On the other hand, the virtual world is able to provide a learning environment with 0% risk to patients even during complex cases. It sounds reasonable to explore these areas in order to enhance skills transfer, and the specialist in training can be adequately educated. Therefore, the study aimed to assess the effectiveness of transferring skills to trainees with a simulation model while patients' risk is minimized in cardiovascular medicine [12].

Objective

This article aims to assess and review studies in the areas of cardiology and vascular medicine in terms of using simulating models and to evaluate the outcome of transferring skills using these simulation training models.

## Review

Design

This is a systematic review of randomized controlled trials on simulation-based training in cardiovascular medicine.

Eligibility criteria

Study Characteristics

This review includes only studies published in the English language from January 2010 to October 2023.

Exclusion Criteria

This study excluded the following:

1. Papers with only abstracts and no access to their full documents.

2. Studies on secondary data, which include non-systematic reviews, narrative reviews, scoping reviews, etc.

Search strategy

Information Sources and Search Strategy

Searches involved several combinations of the search term from medical subject headings (MeSH). Keywords in the title, abstract, and text for the population, intervention, control, and outcomes were first used in a pilot search to establish the sensitivity of the search strategy. Studies were searched in PubMed, Medline, Cochrane Library, Embase, CINAHL, and Hirani.

The search strategy involved the use of medical subject headings, text terms, keywords and words: ("computer simulation"[MeSH Terms] OR ("computer"[All Fields] AND "simulation"[All Fields]) OR "computer simulation"[All Fields] OR "simulation"[All Fields] OR "simul"[All Fields] OR "simulate"[All Fields] OR "simulated"[All Fields] OR "simulates"[All Fields] OR "simulating"[All Fields] OR "simulation s"[All Fields] OR "simulational"[All Fields] OR "simulations"[All Fields] OR "simulative"[All Fields] OR "simulator"[All Fields] OR "simulator s"[All Fields] OR "simulators"[All Fields]) AND ("based"[All Fields] OR "basing"[All Fields]) AND ("education"[MeSH Subheading] OR "education"[All Fields] OR "training"[All Fields] OR "education"[MeSH Terms] OR "train"[All Fields] OR "train s"[All Fields] OR "trained"[All Fields] OR "training s"[All Fields] OR "trainings"[All Fields] OR "trains"[All Fields]) AND ("nat clin pract cardiovasc med"[Journal] OR ("cardiovascular"[All Fields] AND "medicine"[All Fields]) OR "cardiovascular medicine"[All Fields]))

The search applied no search limits.

Study records and data management

Results from the search were imported into Rayyan (Rayyan Systems Inc., Cambridge, MA) to check for duplication of results and consequent duplication. Evaluation and screening of the articles by title, abstract, and full text based on the inclusion and exclusion criteria stated above was also done using Rayyan software. Eligible articles were then selected for the study.

Data Extraction and Management

Article selection and data extraction were performed by the primary reviewer. Results were examined by the second reviewer. Discussion sessions were held by the two reviewers where explanations of all data items and required elements of the study and certification took place. Standardization of the procedure was required for regularity in the method of data extraction used by the reviewers. A third reviewer was consulted when there were disagreements between the primary and secondary reviewers.

**Figure 1:**
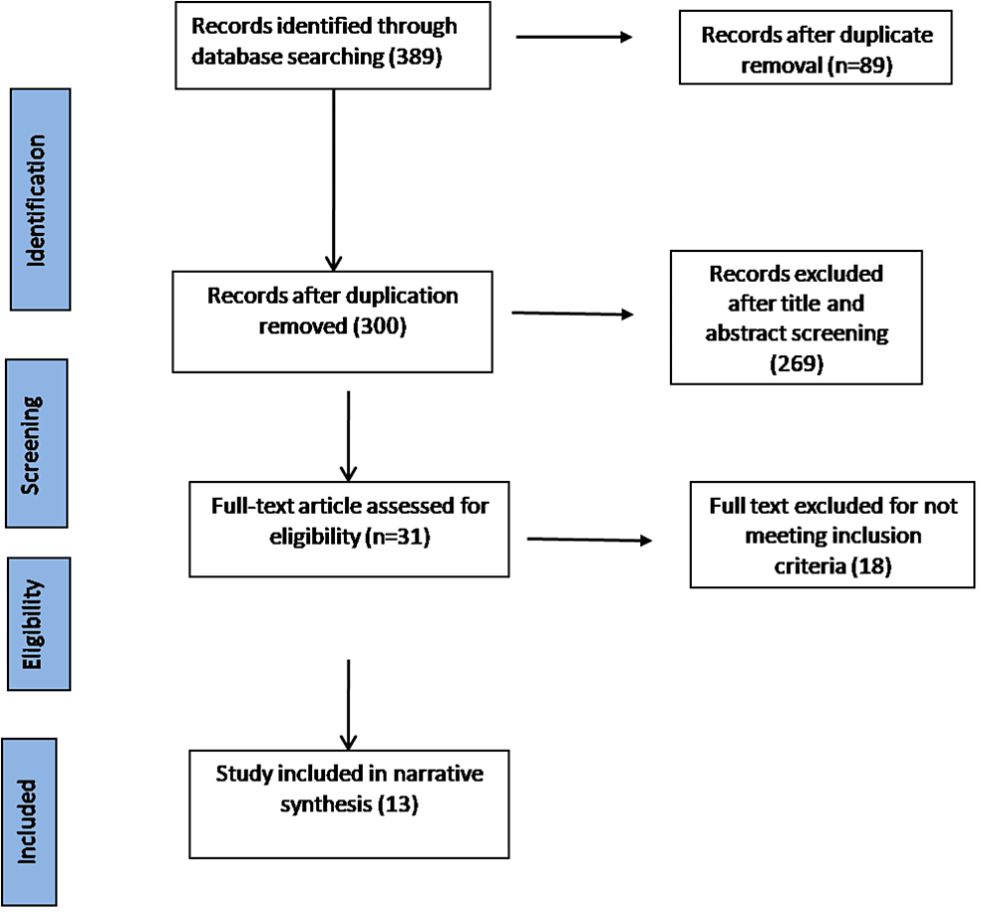
PRISMA flowchart

This method of data extraction is known as single data extraction. Pooling of data was undertaken where adequate homogeneity of results existed. Discrepancies were resolved by a third reviewer. For each included trial, data were extracted regarding the participants (eligibility criteria), the interventions, the control, and the outcome measures. Information on reported benefits and adverse effects was also collected. Details of the screening based on the eligibility criteria along with reasons for exclusion are presented in a flow chart (Figure 1).

Quality appraisal

The quality assessment of the articles was done using the GRADE-CERQual approach tool for systematic review [13]. The GRADE-CERQual approach bases its assessment of confidence on four components: the methodological limitations of individual studies contributing to a review finding; the adequacy of data supporting a review finding; the coherence of each review finding; and the relevance of a review finding. To assess the methodological limitations of the studies contributing data to a review finding, a critical appraisal tool was necessary.

Data items

Data were collected for variables, which included the author’s citation, year, country where the study was carried out, study design, procedure carried out, simulation model, sample size, and outcomes of various studies analyzed.

Risk of bias

The risk of bias was assessed using the Cochrane collaboration tool for accessing risk of bias [14]. This tool comprises six areas of bias: bias of selection, detection bias, performance bias, bias of data loss, reporting bias, and further bias. Eligible studies were assessed and were rated as high risk, low risk, or unclear risk if details reported in the study were not sufficient. Disagreements were discussed and resolved by the entire team.

Data analysis

The analysis and documentation of results were made in tabular format, with the outcomes and additional variables interpreted using narrative synthesis. Studies were interpreted using narrative synthesis following the approved standards by the Center for Reviews and Dissemination to show the relationship and findings between and within the included studies.

Results

A total of 389 studies were obtained from five databases using the search strategies. Eighty-nine studies were excluded for duplication. A total number of 269 studies did not meet the inclusion criteria, excluded based on abstract and title screening, and 18 works excluded were based on full-text screening. In this study, 13 articles were selected ranging from 2011 to 2022. Countries of publications include the United States of America, Canada, Italy, Korea, Ireland, Germany, Belgium, Switzerland, United Kingdom, Netherlands, and France.

Procedures simulated include cardiac catheterization-coronary angiography, transseptal catheterization, cardiopulmonary resuscitation, ultrasound-guided radial artery cannulation, diagnostic angiograms, coiled carotid terminus aneurysms in the setting of subarachnoid hemorrhage, middle cerebral artery embolectomies, renal artery angioplasty/stenting (RAS), endovascular aneurysm repair (EVAR), transvenous pacing (TVP) wire, intra-aortic balloon pump (IABP), and pericardiocentesis (PC).

The simulation models are shown in Table 1 below. Most of the outcomes of the study support that simulation training complements traditional methods of training.

**Table 1:** Data outcomes

Authors	Year	Country	Study design	Procedure	Simulation model	Sample size	Outcome
Lee et al. [10]	2022	United States of America	RCT	Coronary angiography	The Mentice VIST ® -C (Gothenburg, Sweden)	105 (53 Vs. 52 controls)	Simulation training was a good complement to traditional methods as it improved CA interpretation skill
McCoy et al. [15]	2019	United States of America	RCT	Cardiopulmonary Resuscitation	Resusci Anne® CPR manikin	70 (35 Vs. 35 controls)	Improve outcome with simulation training
Bagai et al. [16]	2012	Canada	RCT	Cardiac Catheterization	The MENTICE VIST (Vascular Intervention Simulation Trainer)	27 (12 Vs. 15 controls)	Mentored simulation training was able to transfer skills required to perform cardiac catheterization
De Ponti et al. [17]	2011	Italy	RCT	Transseptal catheterization	the Procedicus VIST (version 7.0, Mentice AB, Gothenburg, Sweden, in cooperation with Biosense Webster, Diamond Bar, California)	14 (7 Vs. 7 controls)	Virtual reality results in shorter training duration of TSP-C as well as superior post-training performance.
Oh et al. [9]	2020	Korea	RCT	ultrasound-guided radial artery cannulation	Blue phantom paediatric four-vessel ultrasound training block model (CAE healthcare®, Sarasota, FL, USA)	21 (11 Vs. 10 controls)	Simulation-based training using a vessel phantom effectively improved the first attempt success rate for ultrasound-guided radial artery cannulation in real patients
Pannell et al. [18]	2016	California	Longitudinal Study	Diagnostic angiograms, coiled carotid terminus aneurysms in the setting of subarachnoid hemorrhage, and middle cerebral artery embolectomies	Simbionix Angio Mentor™	21 (11 Vs. 10 controls)	Trainees across all levels of training and prior experience demonstrated a significant performance improvement after completion of the simulator curriculum
Räder et al. [12]	2014	Canada	RCT	Coronary Angiography	Procedicus virtual interventional simulator trainer [VIST], generic software 8.0.1, Mentice, Gothenburg, Sweden	20 (10 Vs. 10 controls)	The association between CA performance in a simulated setting and actual performance in the catheterization laboratory is not linear. Simulator performance as a predictor of clinical performance should be interpreted with caution.
Boyle et al. [19]	2011	Ireland	RCT	Renal artery angioplasty/stenting (RAS)	Procedicus VIST (Vascular Interventional Surgical Trainer) system [Mentice, Gothenburg, Sweden])	18 (6 control vs 6 expert feedback vs 6 nonexpert feedback)	VR simulator training for novices can significantly improve general performance in the absence of expert trainers
Voelker et al. [20]	2016	Germany	Stratified Randomized Study	Coronary intervention	CoroSimR, Mecora, Aachen, Germany	18 (9 Vs. 9 controls)	curriculum-based mentored VR simulation training improves the performance level of cardiology fellows in coronary interventions
Desender et al. [21]	2017	Belgium, Switzerland, UK, & the Netherlands	A Multicentre Trial	Endovascular aneurysm repair (EVAR)	ANGIO Mentor Express Dual Access Simulation System, Simbionix USA Corp., Cleveland, OH, USA	100 patients	Patient-specific rehearsal (PsR) prior to endovascular aneurysm repair (EVAR) has a significant impact on the treatment plan and may be useful as a pre-operative planning and briefing tool
Popovic et al. [22]	2019	France	RCT	Coronary angiography	Simbionix AngioMentor (Simbionix USA, Cleveland, Ohio)	20 (10 Vs. 10 controls)	Improved operator skills Compared with traditional catheterization laboratory mentor-based training.
Young et al. [23]	2018	USA	Cohort study	Transvenous pacing [TVP] wire, Intra-aortic balloon pump [IABP], and Pericardiocentesis [PC]	State-of-the-art mannequins, and virtual procedural simulation	24 (17 Vs. 7 controls)	Simulation training was shown to be an effective tool to enhance the acquisition of knowledge and technical skills
Prenner et al. [24]	2018	Chicago, USA	RCT	Cardiac catheterization	Procedicus VIST@-C endovascular system	24 (12 Vs. 12 controls)	Simulation-based training is associated with decreased use of fluoroscopy in downstream clinical care during diagnostic coronary angiography

Discussion

In modern clinical practice, the patient-doctor relationship has almost evolved its fiduciary character and has become more formal and structured. The axiom “you learn from your mistakes” is becoming obsolete as medical practice is no longer seen as infallible or beyond questioning; therefore, proficiency in practice is becoming paramount [25]. More so, the training aspect of specialists has gained attention to ensure that medical practitioners are proficient with the required skills and knowledge. Simulation-based training is one of the contemporary methods often used as an adjunct to conventional apprenticeship methods of training, and despite increasing evidence of the benefits of high-quality instruction to trainees, ensuring patient safety and mitigating medical errors, there are still questionable outcomes of simulation-based learning [11,12].

Various positive outcomes have been reckoned with simulation studies. Lee et al. [10] demonstrated that simulation training can be used as a complement to the conventional method, and their findings showed that simulation tutoring has improved coronary angiography skills among trainees. Similarly, McCoy et al. [15] have demonstrated, in a randomized control trial, conducted among medical students where they compared simulation versus standard training for teaching medical students high-quality cardiopulmonary resuscitation. They observed that students in the simulation group performed cardiopulmonary resuscitation that more closely adhered to the American Heart Association guidelines of compression depth and compression fraction. Bagai et al.'s [16] findings from a randomized controlled trial also revealed that skills required to perform cardiac catheterization can be learned via mentored simulation training. These positive findings of learning in the virtual world may be due to the fact that trainees tend to have more access and procedural time in virtual reality than in real life. This is not the case with real-life procedures where modern training is more supervised and targets for clinical tutors lead to a busier operating list with less time for training. In addition, patient safety is of concern. As such, litigations tend to come up when trainees make significant errors, and because outcomes are published in national databases and media, trainees tend to have less exposure to difficult cases.

Despite simulation-based training being a valuable supplemental training resource, it has also suffered a good number of setbacks. It is pertinent to state that with simulation training, one can invariably opine that no matter how accurate the learning simulation is, there is always some room for error and doubt when it comes to the re-creation of real-life scenarios [26]. Although most of the studies reported in this review [9,10,15-24] supported positive outcomes of the simulation-based study, only Räder et al. [12] had a contrary opinion that simulator performance as a predictor of clinical performance should be interpreted with caution. It would be necessary to justify simulation-based training effectiveness with the presence of new and more advanced technology; as such, there is a need to train people to keep up and handle new technology. Therefore training and re-training with different modalities would be important to keep abreast with newer techniques. Additionally, it is important to consider the availability of simulation training facilities as only a few countries have been able to produce and utilize simulation-based training successfully. Lawaetz et al. [27] in their study to explore the status and availability of simulation-based education for learning vascular surgical procedures, have proposed that systematic and structured programs are required to ensure successful implementation. Another setback identified with simulation-based training is the cost and maintenance. Every technology gets outdated with time. Howbeit, with our society that is rapidly evolving, this poses a crucial setback to simulation training as newer technology and update of previous models are essential to effectively practice simulation-based education. Therefore, simulators require regular updates and maintenance based on the changing trends of what is being taught [26]. Despite the accredited drawbacks highlighted, simulation-based training has been shown to offer many advantages compared to traditional teaching methods, as the former has been able to demonstrate abstract concepts into interactive visual content and allows plausible interactions. Studies in this review [9,10,15-24] have proved that simulation studies have improved learning as immediate feedback from procedures helps trainees learn from their mistakes and would be able to carry out procedures in real life with minimal errors and risk to the patient.

Simulation training is gaining ground rapidly [2] in the United Kingdom. The General Medical Council has mandated simulation training for first-year registrars. In the cardiology specialty, skills to perform procedures such as pericardiocentesis and angiography are now incorporated into the simulation training curriculum [27]. Additionally, the East of England training Deanery has incorporated hands-on simulation programs for doctors across the country to practice complicated heart and lung procedures via the Essex Cardiothoracic Centre [28]. This is further supported by the recent introduction (by the Anglia Ruskin University Msc program in medical education) of a whole module option in simulation training education in order to become a trained educator in the simulation environment [29]. Due to the COVID-19 shutdown, the British Society of Echocardiography have considered simulation-based training helpful to continue the training of cardiologists on transoesophageal echocardiography, and they achieve this via simulation software that allows pathology to be well replicated [30]. These examples and application of the new teaching method showed that it is the modus operandi of training in the future if the system is well-structured.

## Conclusions

It will be an added advantage if simulation-based learning is used as an adjunct to the conventional apprenticeship system as several cardiovascular procedures with coherent technicality and tactile control can be demystified without considerable harm to patient care. This study has revealed that simulation teaching reasonably complements traditional training in cardiovascular medicine as skills are successfully transferred to trainees early. Although the availability and cost of maintenance are setbacks to its application, modalities and structures to emphasize its usefulness are essential to its effectiveness. We, therefore, recommend that virtual reality training should be incorporated as a complementary model to the established system of training in the modern cardiovascular curriculum as it will help ensure early skill transfer while guaranteeing patients' safety.
